# Invisibility cloak with image projection capability

**DOI:** 10.1038/srep38965

**Published:** 2016-12-13

**Authors:** Debasish Banerjee, Chengang Ji, Hideo Iizuka

**Affiliations:** 1 Toyota Research Institute of North America, Toyota Motor North America, Ann Arbor, MI 48105, USA; 2 Department of Electrical Engineering & Computer Science, University of Michigan, Ann Arbor, MI 48109, USA; 3 Toyota Central Research & Development Labs., Nagakute, Aichi 480 1192, Japan

## Abstract

Investigations of invisibility cloaks have been led by rigorous theories and such cloak structures, in general, require extreme material parameters. Consequently, it is challenging to realize them, particularly in the full visible region. Due to the insensitivity of human eyes to the polarization and phase of light, cloaking a large object in the full visible region has been recently realized by a simplified theory. Here, we experimentally demonstrate a device concept where a large object can be concealed in a cloak structure and at the same time any images can be projected through it by utilizing a distinctively different approach; the cloaking via one polarization and the image projection via the other orthogonal polarization. Our device structure consists of commercially available optical components such as polarizers and mirrors, and therefore, provides a significant further step towards practical application scenarios such as transparent devices and see-through displays.

Since the pioneering work^1^ that led to the realization of electromagnetic metamaterials over a decade ago, invisibility cloaking of an object to incoming waves has been one of the most appealing possibilities in the metamaterial research community. A scattering cancellation method has been presented for cloaking a subwavelength object^2^. Transformation optics and conformal mapping^1^,^3^,^4^,^5^ can theoretically hide large objects. Following these fundamental concepts, early works have demonstrated cloaking technologies, mostly in narrowband of microwave frequencies^6^,^7^. By reducing to a two-dimensional coordinate transformation, a quasi-conformal mapping approach called ‘carpet cloaking’^8^ has been realized in the microwave^9^, the near infrared^10^,^11^,^12^, and the visible regions^13^. For an ideal cloaking operation these rigorous theories predict the requirement of extreme material parameters and spatial anisotropy of cloaking media. More recently, an optical metasurface cloak to a laser illumination has been realized by manipulating the reflection phase distribution^14^. It is, in general, difficult to implement cloak structures, particularly in the full visible spectrum. Thus, hiding large 3D objects in the full visible wavelength range for practical applications remains a decade-old challenge.

In recent years, it has been possible to fabricate broadband invisibility cloaks in the full visible region when the omnidirectionality requirement is abandoned. In these cases, very promising results have been obtained when the transformation optics was simplified by removing the design restriction of phase preservation. A polygonal cloak structure made of calcite, which is a natural anisotropic material, was shown to hide a cylindrical object with a diameter of a few millimeters^15^, following the study of calcite carpet cloaks^16^,^17^. In ref. 18, a large object was concealed in normal incidence by using a cloak structure consisting of isotropic material lenses. A number of authors have reported various cloak structures consisting of conventional optical components in the visible region^19^,^20^,^21^,^22^,^23^.

On the other hand, there is a tremendous amount of information such as texts, images, and movies in our daily lives owing to the significant achievements in the image projection technology^24^,^25^,^26^. Modern human vision systems such as 3D displays rely on several psychological cues where overlapping of images, shading, textured gradients are used to create various depth perceptions in the human brain. However, these image-based devices are often associated with ambiguities and errors because of the inability to correctly carry the depth profile information. It is suggested in ref. 24 that in order to achieve the perfect 3D display technology, some novel methods to generate physiological cues such as binocular display, convergence, and accommodation are necessary, which to be combined in the imaging system with existing psychological cues described earlier. To create physiological cues, modern displays often utilize optical elements such as lenticular lenses, parallax barriers, curved lens arrays, holography, and see-through display technologies. However, the aspect of optical invisibility cloaking in such imaging systems has never been explored. For example, the combination of the cloaking with the imaging systems can allow an additional degree of freedom for the physiological cues such as used for the realization of see-through 3D displays and augmented reality technologies^27^,^28^ that could be beneficial to the thrust for transparent electronics in the future.

In this Letter, we experimentally demonstrate an invisibility cloak where a large object can be concealed in the cloak structure and any images can be projected on it simultaneously. This unique functionality can be obtained through the use of one polarization of light for the cloaking and the other orthogonal polarization for the image projection due to the insensitivity of human eyes to the polarization. Such approach has never been captured by any other reports in the past.

## Results

### Mechanism of our invisibility cloak with the image projection capability

**Figure 1:**
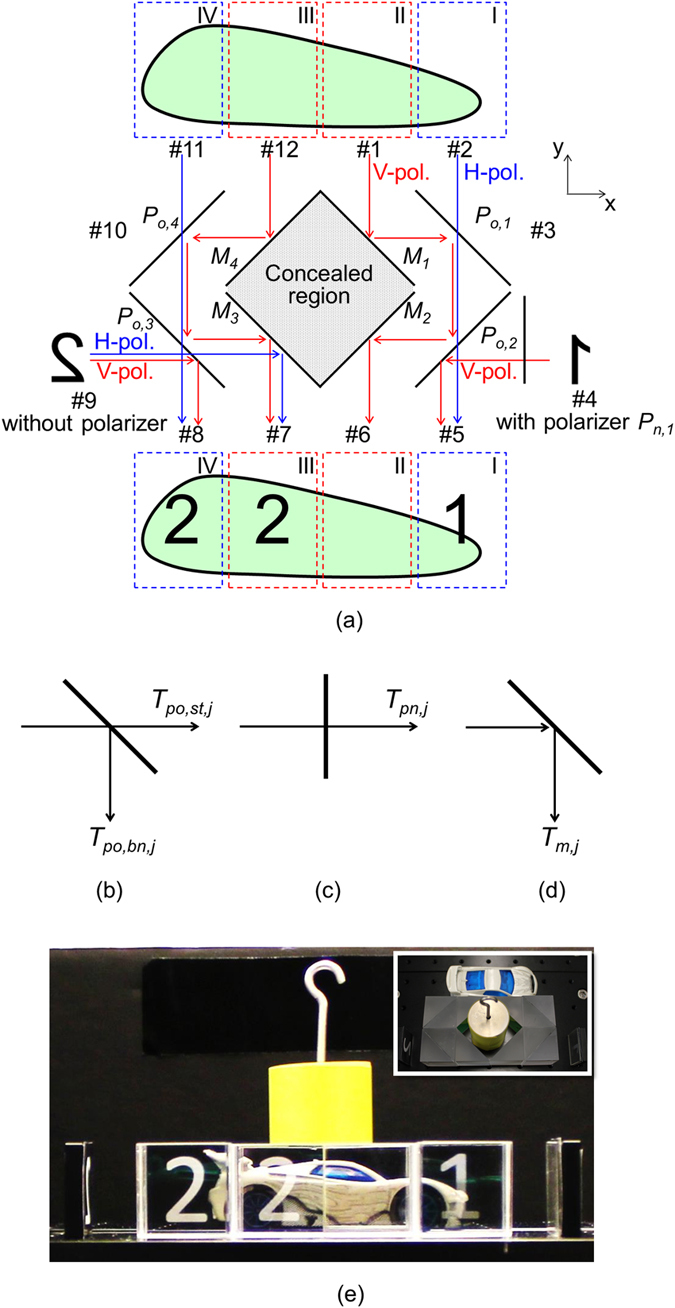
(**a**) Configuration of a cloak structure having the image projection capability. The structure consists of polarizers for oblique incidence (*P*_*o,1*_-*P*_*o,4*_), mirrors (*M*_*1*_-*M*_*4*_), and a polarizer for normal incidence (*P*_*n,1*_), where port 4 has the polarizer *P*_*n,1*_, and port 9 does not have a polarizer for comparison. Labels of transmission coefficients of (**b**) a polarizer for oblique incidence, (**c**) a polarizer for normal incidence, and (**d**) a mirror for the use in Table 1. (**e**) Experimental observation of the cloak structure. The inset shows the experimental setup. A toy car is placed behind the cloak structure and a cylindrical object is partly inserted in the cloak structure. The white number “1” or “2” printed on a black paper reversely in the left and right is attached on each lateral side, port 4 or port 9.

Our cloak structure consists of commercially available optical components; polarizers for oblique incidence (*P*_*o,1-4*_) and for normal incidence (*P*_*n,1*_), and mirrors (*M*_*1-4*_), as shown in Fig. 1(a). We regard our structure as a twelve-port device and each port is labeled by 1 to 12. In the description of the mechanism of our structure below, we consider that the scene behind the cloak structure is built at the observation point in front of the cloak structure, assuming ideal performance of each component. The regions of the scene are labeled by I to IV, and here we assume that regions I and IV (II and III) are observed via the horizontally (vertically) polarized light. Due to the symmetry of the cloak structure, we consider here the half of the structure for the explanation of the cloak mechanism. To illustrate the image projection capability, one lateral side (port 4) has the polarizer *P*_*n,1*_ and the other lateral side (port 9) does not have a polarizer for comparison.

Consider first the unpolarized light traveling in the −*y* direction from region II of the scene behind the cloak structure in Fig. 1(a). The light is reflected by mirror *M*_*1*_ into the +*x* direction. Here we assume that at ideal obliquely incident polarizers *P*_*o1-4*_, the vertically polarized light is bent by 90° (*T*_*po,bn,V*_ = 1 and *T*_*po,st,V*_ = 0 in Fig. 1(b)) and the horizontally polarized light passes straight through (*T*_*po,bn,H*_ = 0 and *T*_*po,st,H*_ = 1 in Fig. 1(b)). Thus, the vertically polarized light is bent twice at *P*_*o,1*_ and *P*_*o,2*_ and then comes out at port 6 via the reflection at mirror *M*_*2*_. Likewise, for the unpolarized light coming from region I, the horizontally polarized light goes through the two polarizers *P*_*o,1*_ and *P*_*o,2*_ and comes out at port 5. Other orthogonal polarized lights coming from areas I and II go out at port 3 (light paths are not presented). In other words, the scene behind the cloak structure is built at the observation point through the use of 50% incidence.

In our structure, the capacity for another 50% light is used for the image projection. The information sources “1” and “2” are placed at ports 4 and 9, respectively, which are reversed in the left and right due to the mirror reflection. Consider the light paths from port 9. The horizontally polarized light goes through polarizer *P*_*o,3*_ and is reflected by mirror *M*_*3*_ and then goes out at port 7 while the vertically polarized light comes out at port 8 by a 90° bent at the polarizer *P*_*o,3*_. As a result, we observe “2” in both regions III and IV. On the other hand, from port 4, we observe “1” in only region I by inserting polarizer *P*_*n,1*_ that allows the transmission of the vertically polarized light. Therefore, the information can be projected in any region(s) as desired. Table 1 shows the summary of light paths with labels of ideal components defined in Fig. 1(b–d). We comprehend the mechanisms of the cloaking and the image projection presented above.

### Experimental demonstration of the invisibility cloak with the image projection capability

The experimental setup is shown in the inset of Fig. 1(e). Our cloak was implemented with four wire-grid polarizer cubes (89–604, Edmund Optics) and four right angle mirrors (45–595, Edmund Optics). White numbers “1” and “2” are printed on black papers reversely in the left and right, respectively, and these papers are placed at lateral sides. A wire-grid polarizer film (47–102, Edmund optics) is attached on the black paper having “1”, where the vertically polarized light passes through. A cylindrical object is placed within the cloak structure, where the cylindrical object is partly in the concealed region while remaining upper part is exposed outside the cloak structure. There is a toy car behind the cloak structure and we capture screen shots through a camera in front of the cloak structure. Figure 1(e) shows an experimental observation of the cloak structure from the camera. The cylindrical object becomes invisible in the cloaking area and the car behind the cloak structure is observed. We observe “1” in region I and “2” in regions III and IV, as we designed. Therefore, the mechanism of our invisibility cloak with the image projection capability has been experimentally verified for human eyes.

### Performance of the image projection capability

**Figure 2:**
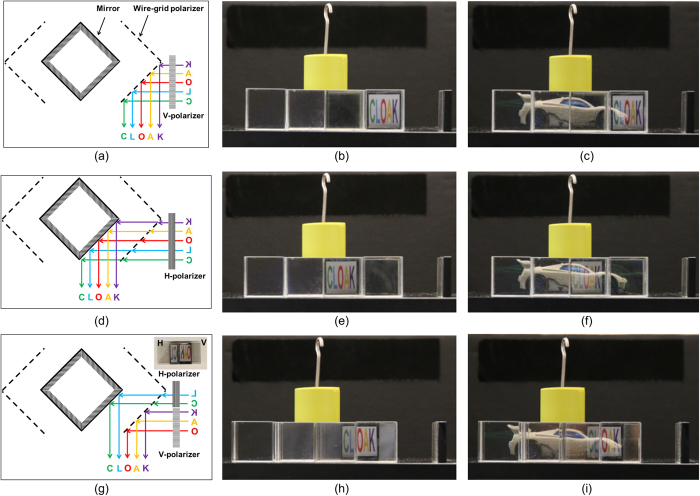
Image projection ability. The text “CLOAK” appears in region I for (**a–c**), in region II for (**d–f**), and in the middle of regions I and II for (**g–i**). Light paths (**a**,**d**,**g**), the image projection (**b**,**e**,**h**), and the cloaking (**c**,**f**,**i**) are presented.

We further investigate the image projection ability, particularly, the placement of the image projection. We show that an image can be projected at the middle of neighboring regions as well as each region. As expected from the experimental demonstration of Fig. 1(e), the text “CLOAK” appears at region I (Fig. 2(b)) by selecting the vertical polarization (Fig. 2(a)), which is directly reflected by the side polarizer *P*_*o2*_, and appears at region II (Fig. 2(e)) with the horizontally polarized light going through the polarizer *P*_*o2*_ and being reflected by the mirror *M*_*2*_ instead (Fig. 2(d)). In this experimental demonstration, the wire-grid polarizer film (47–102, Edmund optics) was rotated by 90° for the polarization change. The text “CLOAK” can be projected at the middle of regions I and II (Fig. 2(h)) via the horizontally polarized light for “CL” and the vertically polarized light for “OAK” (Fig. 2(g)). Therefore, proper use of polarizers for an image enables the projection in any places on the cloak structure while the invisibility cloak phenomenon is maintained (Fig. 2(c,f,i)).

### View angle dependence

**Figure 3:**
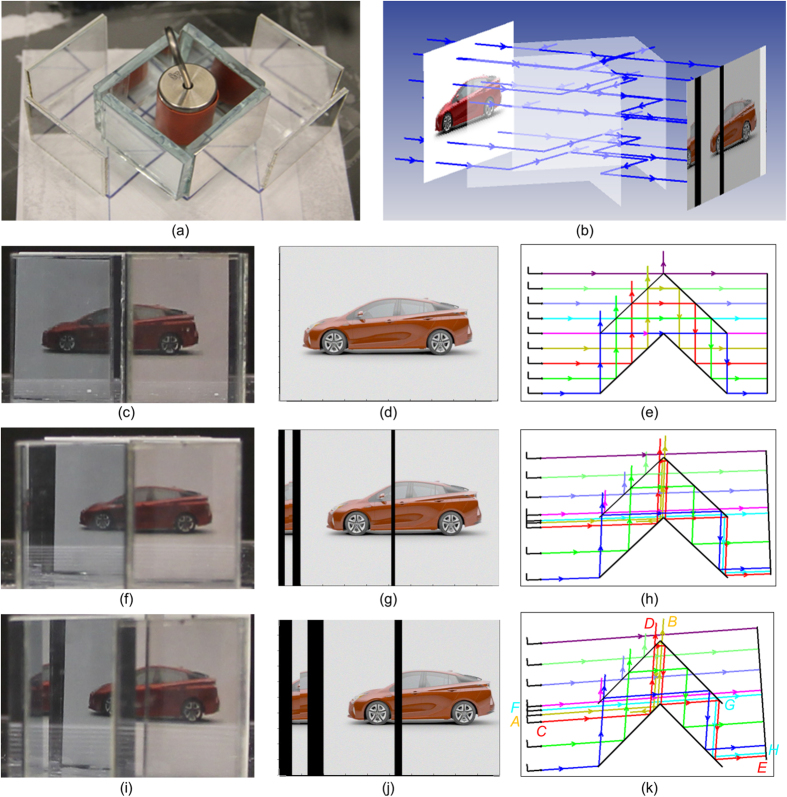
View angular dependence of the cloak structure. (**a**) Experimental setup. (**b**) Simulation setup. Experimental observations of the scene including a red car ((**c**,**f**,**i**)), the simulation results ((**d**,**g**,**j**)), and the corresponding light paths ((**e**,**h**,**k**)) are presented at view angles of 0° ((**c**–**e**)), 2° ((**f–h**)), and 4° ((**i**–**k**)), respectively.

The dependence of our structure on the view angle is experimentally and numerically investigated and shown in Fig. 3. To illustrate the main reason for the degradation of the rebuilt scene with the increase of the view angle, the cloak structure now consists of polarizers and mirrors that are attached on glass plates (Fig. 3(a)), instead of glass blocks. This allows us to envision what happens in the cloak structure by optical paths clearly without the effect of the refractive index of glass. The half of the structure is used for this investigation due to the symmetry of our cloak structure; the scene including a car has the same size as the summation of regions I and II. Optical paths from the original scene are calculated and the scene rebuilt at the observation plane is obtained (Fig. 3(b)) by using the commercial optical design software Zemax^29^. The view angle has been set at 0°, 2°, and 4°, respectively. In the normal direction, the rebuilt scene is same as the original scene except for the half of the brightness (Fig. 3(d)) due to the use of 50% light (Fig. 3(e)). As the view angle increases, the rebuilt image is degraded manifesting as the lack of the information (black lines in Fig. 3(g,j)) and the wrong position of the information (the middle part of the red car appears at the left edge in Fig. 3(g,j)), respectively. These can be analyzed and well understood from optical paths tracing as shown in Fig. 3(e,h,k) for different incident angles. Taking the 4° incidence for example, light path *A-B* (yellow color ray) represents one source of image information lacking as the light ray is reflected to the neighboring wire-grid polarizer *P*_*o2*_ directly at the oblique incidence. On the other hand, the wrong position of the output image information is credited to the rays near *F-G-H* path (highlighted as the cyan color), where unpolarized light is directly incident onto the wire-grid polarizer at large angles of incidence and then split into two different paths with one path going to the correct position while the other to the left edge. It is worthwhile to mention that there is another source represented by and near *C-D-E* (red color) for both the two image degradation results. This portion of light is completely misled to the output edge leaving a black line at the center. Note that the lack and the wrong position of the information mentioned above are consistent with the cloak structure of Fig. 1(e).

## Discussion

In recent times, modern electronic devices such as cellphones, smart glasses, and computers are desired to be transparent^30^. The need for transparent objects with the display function is not limited to personal electronics. Tremendous applications have also been envisioned from the automotive industry, household appliances to healthcare sector. Typically, two approaches are adopted to realize transparent devices; firstly, a quest for materials with the unusual combination of high electrical conductivity and high optical transparency such as Indium Tin Oxide, and the composites of carbon nanotubes, graphene, and silver nanowires^31^. Despite the recent significant progress, mass application of these materials has remained challenging. On the other hand, driven by the desire to develop augmented reality experience, transparent or see-though displays are aimed to project virtual images of 2D or 3D objects utilizing optical components such as wedge-shaped prisms, spherical mirrors, array of lenses, etc.^32^. Holography based optical elements have also been used^33^. However, as we discussed before, optical cloaking approaches combined with the imaging systems have not been explored in the past. In our experiment, we have used printed papers as information sources for the experimental demonstrations in order to verify the mechanism and architecture of the display aspect in combination with cloaking the central part of the device. In practical applications, one can implement two thin-type displays having electrically switchable polarizers at ports 4 and 9 and project text messages and movies at any locations of the cloak structure. We believe that combining the invisibility cloaking with the image projection capability promises an alternative route to realize next generation transparent devices.

In conclusion, we have explored a cloak structure that provides the image projection capability. Due to the insensitivity of polarization for human eyes, the cloaking and the image projection are simultaneously obtained via the use of orthogonal polarizations in our structure. The mechanism of our structure was experimentally verified for human eyes. Our cloak structure consists of commercially available optical components and provides a significant further step towards practical applications in see-through displays and electronics.

## Additional Information

**How to cite this article**: Banerjee, D. *et al*. Invisibility cloak with image projection capability. *Sci. Rep.* **6**, 38965; doi: 10.1038/srep38965 (2016).

**Publisher’s note:** Springer Nature remains neutral with regard to jurisdictional claims in published maps and institutional affiliations.

## Figures and Tables

**Table 1:** Transmission coefficients of the cloak structure of Fig. 1(a).

	Input port						
		1	2	3	4	5	6
Output port	1	—	0	*T*_*po,st,j*_*T*_*m,j*_ (0,1)	0	*T*_*po,bn,j*_*T*_*po,st,j*_*T*_*m,j*_ (0,0)	*T*_*po,bn,j*_^*2*^*T*_*m,j*_^*2*^(1,0)
	2	0	—	*T*_*po,bn,j*_ (1,0)	0	*T*_*po,st,j*_^*2*^(0,1)	*T*_*po,st,j*_*T*_*po,bn,j*_*T*_*m,j*_ (0,0)
	3	*T*_*po,st,j*_*T*_*m,j*_ (0,1)	*T*_*po,bn,j*_ (1,0)	—	0	0	0
	4	0	0	0	—	*T*_*po,bn,j*_*T*_*pn,j*_ (1/0,0)	*T*_*po,st,j*_*T*_*pn,j*_*T*_*m,j*_ (0,1/0)
	5	*T*_*po,bn,j*_*T*_*po,st,j*_*T*_*m,j*_ (0,0)	*T*_*po,st,j*_^*2*^(0,1)	0	*T*_*po,bn,j*_*T*_*pn,j*_ (1/0,0)	—	0
	6	*T*_*po,bn,j*_^*2*^*T*_*m,j*_^*2*^(1,0)	*T*_*po,st,j*_*T*_*po,bn,j*_*T*_*m,j*_ (0,0)	0	*T*_*po,st,j*_*T*_*pn,j*_*T*_*m,j*_ (0,1/0)	0	—

The cloak structure is regarded as a twelve-port device and transmission coefficients for six ports are presented due to the mirror symmetry of the structure. Each coefficient in the table is defined in Fig. 1(b–d). Parentheses (*T*_*j=V*_,*T*_*j=H*_) represent transmittances for vertical polarization and horizontal polarization, assuming ideal performance of each component (*T*_*m,j*_ = 1, *T*_*po,st,j*_ = 1 or 0, *T*_*po,bn,j*_ = 1 or 0, and *T*_*po,st,j*_*T*_*po,bn,j*_ = 0). 1/0 represents that the transmittance can be changed by polarized light.
